# Artesunate – amodiaquine combination therapy for falciparum malaria in young Gabonese children

**DOI:** 10.1186/1475-2875-6-29

**Published:** 2007-03-12

**Authors:** Sunny Oyakhirome, Marc Pötschke, Norbert G Schwarz, Jenny Dörnemann, Matthias Laengin, Carmen Ospina Salazar, Bertrand Lell, Jürgen FJ Kun, Peter G Kremsner, Martin P Grobusch

**Affiliations:** 1 Medical Research Unit, Albert Schweitzer Hospital, Lambaréné, Gabon; 2 Department of Parasitology, Institute of Tropical Medicine, University of Tübingen, Tübingen, Germany; 3 Infectious Diseases Unit, Division of Clinical Microbiology and Infectious Diseases, National Health Laboratory Service and School of Pathology, Faculty of Health Sciences, University of the Witwatersrand, Johannesburg, South Africa

## Abstract

**Background:**

Artesunate-amodiaquine combination for the treatment of childhood malaria is one of the artemisinin combination therapies (ACTs) recommended by National authorities in many African countries today. Effectiveness data on this combination in young children is scarce.

**Methods:**

The effectiveness of three daily doses of artesunate plus amodiaquine combination given unsupervised (n = 32), compared with the efficacy when given under full supervision (n = 29) to children with falciparum malaria were assessed in an unrandomized study.

**Results:**

61 patients analysed revealed a PCR-corrected day-28 cure rate of 86 % (25 of 29 patients; CI 69 – 95 %) in the supervised group and 63 % (20 of 32 patients; CI 45 – 77 %) in the unsupervised group. The difference in outcome between both groups was statistically significant (p = 0.04). No severe adverse events were reported.

**Conclusion:**

The effectiveness of this short course regimen in young children with falciparum malaria could be augmented by increased adherence and improved formulation.

## Background

Antimalarial chemotherapy has been the primary option in the fight against malaria. However, the burden of this disease is still very heavy partly due to the development of multi-drug resistant *Plasmodium falciparum *strains [1-4]. The malaria community presently considers mono-therapy as an inappropriate approach for malaria treatment[5]. African countries have recently begun to scale up their antimalarial efforts, and are deploying strategies to combat the new face of malaria. One of these strategies is the use of artemisinin-based combination therapies (ACTs) which have proven to be very effective against malaria in Africa, and some African countries plagued with resistant forms of *P.falciparum *have started instituting the ACTs as first line malaria treatment [6,7].

Artesunate plus amodiaquine combination is one ACT recommended by the World Health Organization (WHO) for use in malaria control programmes and a first line treatment for African children with uncomplicated malaria [8]. This recommendation is now a national policy in Gabon.

Artesunate and amodiaquine are generally safe and well tolerated when used as treatment for malaria [9-18]. In a previous study the efficacy of artesunate-amodiaquine for uncomplicated *P. falciparum *malaria in Gabonese children was 94% [19], but the effectiveness of this combination under outpatient conditions in Gabon is not known.

There have been very few effectiveness studies on ACTs. Depoortere et al. [20] found that patients who received a combination of sulphadoxine/pyrimethamine and artesunate under supervision achieved a 28-day PCR corrected cure rate of 84% and effectiveness rate of 63%. The critical reader of the paper by Piola and colleagues [21] will find that despite final cure rates of 98% and 97% in both supervised and unsupervised subjects, there were substantial differences in lumefantrine blood concentrations in patients from the two groups, indicating that sometimes a difference between the efficacy and effectiveness of a highly efficacious drug like artemether-lumefantrine may not be demonstrable. It is important, therefore, that effectiveness of drugs is measured alongside clinical efficacy, considering that under everyday conditions many drug combinations do not reach the same cure rates that were measured in clinical trials under ideal conditions and supervised drug intake.

In the present study, we assessed the effectiveness of three-day artesunate plus amodiaquine combination administered unsupervised, compared with the efficacy when given under supervision to children with *P. falciparum *malaria was assessed.

## Methods

### Study site

This study was conducted a study in Lambaréné from July 2004 to June 2005 on a cohort of children participating in an ongoing trial at the Medical Research Unit of the Albert Schweitzer Hospital (HAS), Gabon. Lambaréné is a small town of approximately 30,000 inhabitants located near the equator in the Central African rainforest belt. Malaria transmission is moderate and perennial. 95% of all malaria infections are caused by *P. falciparum *and the entomological inoculation rate is about 50 infective bites per person per year [22,23].

### Study population

The study subjects are a subgroup of participants in a prospective Intermittent Preventive Treatment intervention (IPTi) trial [24,25] – administering treatment doses of sulphadoxine – pyrimethamine (or placebo) to children at three, nine and 15 months and following them up monthly for 30 months.

All participants of the IPTi trial were included who had uncomplicated malaria, defined as the presence of asexual parasitaemia of *P. falciparum *with a rectal temperature of at least 38.5 °C or a history of fever in the last 48 hours. Written or documented oral consent of the parents or the guardians of the children were obtained at enrolment into the main study, usually after birth in the maternity ward of the Albert Schweitzer Hospital or the Public Regional Hospital in Lambaréné. Ethical clearance was obtained from the Ethics Committee of the International Foundation of the Albert Schweitzer Hospital in Lambaréné.

Two groups of children were compared; those that received artesunate plus amodiaquine combination under supervision and those who received the combination unsupervised. Assignment of the two groups was not randomized. According to the IPTi protocol, all study subjects with falciparum malaria were treated with three daily doses of artesunate plus amodiaquine combination from July 2004. The first dose was administered at the Medical Research Unit and the second and third doses at home. This treatment was classified as unsupervised. Then, from December 2004 all patients received all three doses of artesunate-amodiaquine combination under supervision at the Medical Research Unit. This treatment was classified as supervised.

All cases that had (i) a mixed infection or (ii) received a sufficient dose of another antimalarial drug a week prior or during the 28-day post-malaria follow-up period or (iii) taken the IPTi study medication a week prior or during the 28-day post-malaria follow-up period were excluded from intention to treat (ITT) analysis. If they vomited the artesunate plus amodiaquine combination twice, they were excluded from the evaluability analysis.

### Study procedure

Treatment consisted of a daily oral dose of artesunate 4 mg/kg body weight plus amodiaquine 10 mg/kg body weight given for three days (ARSUCAM™ provided by Sanofi Synthélabo). Artesunate and amodiaquine were supplied in tablets which were crushed then mixed with sugar into syrup and given orally. A full dose of artesunate-amodiaquine combination was re-administered after 30 minutes if the child either spat the medication out or vomited within one hour.

The day of treatment was considered as day 0. Patients were invited for follow up visits on days 2 and 28. A clinical and laboratory assessment was done on these follow up visits. These included a thick blood smear and a full blood count. The Giemsa-stained thick smears which were read by at least two experienced microscopists. Parasitaemia was quantified (number/μL) by the Lambaréné method [26]. A smear was declared negative only after ≥100 visual fields were scrutinized.

We advised the parent or guardian to administer doses as seen or practiced with the first dose, and to report back to the hospital if the child vomited or refused to take the medication. We also encouraged the parents or guardians to return back to the hospital at any time in case the child's health appeared to deteriorate.

### Definition of study end points

Our primary outcome was defined as a parasitological cure on day 28. Failure was defined as persistent parasitaemia or reappearance of parasites during the follow-up period of 28 days. Our secondary outcome was the safety of the artesunate-amodiaquine combination drug measured as the frequency of adverse events. These were defined as any signs or symptoms or any abnormal laboratory value not present on day 0 or becoming worse during follow up and were judged by the clinicians in the study with respect to severity and relationship to study drug.

### Full blood count

We measured the haemoglobin (Hb), white blood cell count (WBC) and neutrophils count on days 0 and 28 with an automated analyser (Cell-Dyn 3000™, Abbott Diagnostics Santa Clara, CA).

### MSA-1 and MSA-2 genotyping

Filter-paper blots (Glass fibre filters: Schleicher & Schuell MicroScience, Dassel, Germany) were taken on day 0 and on the day of recurrent parasitaemia for polymerase chain reaction (PCR). For optimal differentiation between strains, and as described before [27,28], we genotyped parasites for merozoite surface antigens MSA-1 and MSA-2 as two non-linked genotypical markers, in order to distinguish between re-infection and recrudescence in the case of reappearance of parasites during the follow-up period of 28 days.

### Data analysis

Efficacy was assessed by evaluability (according-to-protocol, ATP) and ITT (intention-to-treat) analysis. Cure rates were calculated from the number of patients with clinical and parasitological cure by day 28 divided by total number of patients in the ITT population or per protocol population respectively. The according-to-protocol population was defined as children who completed the 3-day regimen of daily artesunate plus amodiaquine and had a day 28-follow up visit (+/- 1 week). There were 61 cases eligible for ATP analysis. The intention-to-treat population was defined as children who took at least the first dose. There were 89 cases eligible for ITT analysis. Based on a previous randomized trial of artesunate plus amodiaquine in our study area [18], a PCR-corrected day-28 cure rate of 90 % in the observed group was assumed.

Any data inconsistencies were reconciled, and the data were analyzed with the statistical software JMP 5.0 and Stata 8.2 (StataCorp, Texas, USA). A descriptive analysis of both observed and unobserved group was made, and continuous data (age, temperature and haemoglobin concentrations) between groups by unpaired t-test and categorical variables with a chi-square test were compared.

## Results

**Figure 1:**
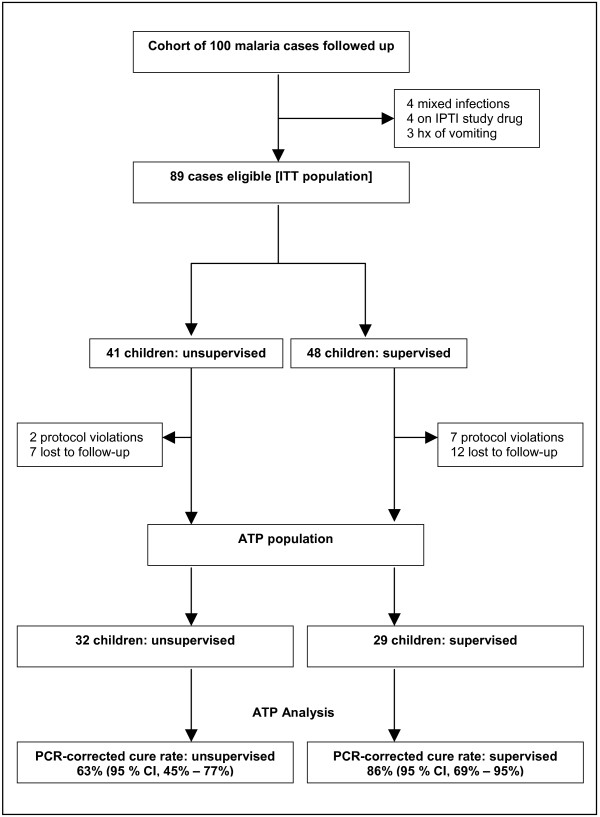
Trial profile.

Figure 1 depicts the trial profile. Baseline characteristics of study subjects are given in table 1. In the ATP analysis of this cohort of 61 patients, the proportion of children cured in the supervised group was 86% (25/29, 95% CI; 69% – 95%) and 63% (20/32, 95% CI; 45% – 77%) in the unsupervised group (p = 0.04) (Table 2). Intention-to-treat analysis yielded a similar decrease in proportion cured; 63% (30/48, 95 % CI; 48 – 75%) in the supervised group and 49% (20/41, 95 % CI; 34% – 63%) in the unsupervised group (p = 0.2).

**Table 1:** Baseline characteristics

ATP population n	Total 61	supervised 29	unsupervised 32	p-value
Number of participants (n)	61	29	32	
Age (months)	61	15.1 (6.1)	14.0 (4.9)	p = 0.3
Haemoglobin (g/dl)	53	8.7 (1.5)	8.1 (1.0)	p = 0.2
Temperature (°C)	60	38.4 (1.2)	38.8 (1.2)	p = 0.3
Neutrophils (k/μl)	48	2.8 (2.4)	3.1 (1.8)	p = 0.6
Splenomegaly (n)	48	6.0 (12.5%)	10.0 (20.8%)	

Data are mean (SD) or number (%).

**Table 2:** Day-28 cure rates

ATP population n	total 61	supervised 29	unsupervised 32	p-value
Cure rate (PCR uncorrected)	44/61 (72%)	25/29 (86%)	19/32 (59%)	0.02
Cure rate (PCR corrected)	45/61 (74%)	25/29 (86%)	20/32 (63%)	0.04

The risk ratio in the ATP analysis indicates that supervised administration of artesunate plus amodiaquine combination to children aged below 30 months have a lower risk of failure to treatment compared to when unsupervised (risk ratio= 0.38, p = 0.04). Supervised administration of artesunate plus amodiaquine combination to children aged below 30 months is also positively associated with an increase in probability of achieving a cure from uncomplicated malaria (risk ratio of 1.4 and 1.2 respectively in the ATP and ITT analysis). This translates into an increase of 27% benefit if administration of this drug is supervised. Equivalently, one more cure with artesunate plus amodiaquine can be achieved for every 4 patients treated under supervision.

The median parasitaemia of our cohort was 7,500 parasites/μL. The mean haemoglobin concentration in the supervised and unsupervised groups on day 0 were 8.7 g/dL and 8.1 g/dL respectively (p = 0.16), and on day 28 were 9.5 g/dL and 8.9 g/dL respectively (p = 0.03). The mean changes were not different between the groups. However, on day 0, thirty-nine patients (64%) had anemia defined as haemoglobin between 5 and 9 g/dL. On day 28, eighteen patients (30%) fell under this definition of anemia. Overall the mean haemoglobin concentration increased from 8.4 g/dL on day 0 to 9.2 g/dL by day 28.

There were 36 adverse events reported during the course of this study, the most reported were cough (18%), diarrhoea (12%), vomiting (5.6%), and skin infections (4.5%). There was no serious adverse event or death recorded. In the supervised group, two subjects were treated with the five-day artesunate monotherapy [29], because they vomited after a repeated dose of artesunate plus amodiaquine. Three subjects missed the second dose, one missed the third dose and 1 missed the second and third dose, while in the unsupervised group, two subjects were hospitalized for weakness. These nine protocol violations were excluded from the ATP analysis.

## Discussion

This study shows that supervised administration of artesunate plus amodiaquine to Gabonese children aged less than 30 months with uncomplicated falciparum malaria substantially reduces the risk of treatment failure compared to unsupervised administration. The proportion of supervised children cured by day 28 was 86%, while that of unsupervised children was substantially lower (63%). In a previous randomized study carried out in Lambaréné, supervised administration of artesunate plus amodiaquine achieved a PCR corrected cure rate of 94% by day 28 [19]. The difference in proportion cured when supervised and when unsupervised does not only reflect the significant gap between the usual optimistic efficacy reports of studies and the real-life situation [20], but puts question on the effectiveness of this combination in the treatment of young children with falciparum malaria especially under the usual unsupervised outpatient condition.

Artesunate plus amodiaquine combination was well tolerated. There were no serious adverse events reported during the course of this study. The weakness of our study was that it was not randomized and the sample size was relatively small. Crushed tablets that are probably harder to administer than palatable syrup were used. There is a clear benefit of supervised compared to an unsupervised treatment shown in the ATP analysis which was less evident in the ITT analysis. Certainly the effectiveness of 63% under unsupervised conditions in this study is unacceptably low. This effectiveness might be higher with a fixed combination better tasting syrup given under supervised conditions.

## Conclusion

The therapeutic life of this first line antimalarial recommended for the treatment of uncomplicated *P.falciparum *malaria [30,31] can be maintained. The effectiveness of artesunate plus amodiaquine for treatment of falciparum malaria in Lambaréné will depend on how well adherence to this regimen can be ensured. The present plan of Drugs for Neglected Diseases Initiative (DNDi) [32] and partners to make this fixed-dose of artesunate-amodiaquine available in 2006, calls for more effectiveness data on this combination. This will guide policy implementation that will assist malaria control programmes and clinics to develop strategies that encourage adherence to this regimen, which in turn will prolong the effective life span of this combination for the treatment of falciparum malaria [20,33].

## Authors' contributions

SO designed the study, collected data and prepared the manuscript.

MP designed the study, collected data and prepared the manuscript.

NGS contributed in the preparation of the manuscript.

JD, ML and COS collected data and participated in the study design.

JFK contributed to the molecular genetic studies and contributed to draft the manuscript.

BL, PGK and MPG helped in designing the study and in data analysis and interpretation.
